# Nursing students’ knowledge regarding sexuality, sex, and gender diversity in a multicenter study

**DOI:** 10.3389/fpsyg.2024.1267280

**Published:** 2024-03-12

**Authors:** Conceição Santiago, Açucena Guerra, Teresa Carreira, Sara Palma, Florbela Bia, Jorge Pérez-Pérez, Ana Frias, Sagrario Gómez-Cantarino, Hélia Dias

**Affiliations:** ^1^Higher School of Health, Santarém Polytechnic University, Santarém, Portugal; ^2^CINTESIS, University of Porto, Porto, Portugal; ^3^Nursing Department, Lisbon University, Lisbon, Portugal; ^4^Nursing Department, Higher School of Nursing São João de Deus, Évora University, Évora, Portugal; ^5^Nursing Department, Catholic University of Portugal, Lisbon, Portugal; ^6^Faculty of Physiotherapy and Nursing, University of Castilla-La Mancha, Toledo, Spain; ^7^Comprehensive Health Research Centre (CHRC), University of Évora, Évora, Portugal; ^8^Health Sciences Research Unit: Nursing (UICISA: E), Coimbra Nursing School (ESEnfC), Coimbra, Portugal

**Keywords:** sexuality, sex, gender diversity, sexual knowledge, nursing students

## Abstract

**Introduction:**

Sexuality is an integral part of development and personality, and is important in healthcare. Nurses are among the most representative healthcare professionals. For holistic and inclusive nursing care practice and to improve equality, human rights, well-being, and health of individuals, the curricula of nursing courses must integrate broad knowledge about sexuality and its diversity. This study aimed to identify and analyze nursing students’ knowledge of sexuality, sex, and gender diversity. The present study was part of a multicenter study conducted in Europe.

**Methods:**

Questionnaires were administered in three nursing schools to assess nursing students’ knowledge (n = 75). Data processing was performed using Excel® software version 20 and IRaMuTeQ (R Interface pour les Analysis Multidimensionnelles de Textes et de Questionnaires) 0.7 alpha 2, allowing organization by category and subsequent thematic analysis using content analysis.

**Results:**

The *textual corpus* “Nursing students’ knowledge about sexuality in its diversity,” was divided into two sub-corpus: “Students’ perception of sexuality” and “Students’ perception of gender identity,” originating Class 6 “Eroticism” (14.23%) and Classes 4 “Sexual Orientation” (16.07%) and 3 “Heteronormative” (16.07%), the latter with greater proximity to each other and consequently to Class 6. Similarly, Classes 1 “Gender” (20.36%) and 5 “Cisgender” (12.14%) also presented a greater interrelationship between themselves and consecutively with Class 2 “Gender Identity” (15.36%).

**Discussion:**

The analyses revealed that though nursing students possessed knowledge about sexuality and its diversity, this knowledge was elementary and did not reveal a sustained appropriation of concepts related to sexuality, sexual orientation, and gender diversity. For some questions, the absence of students’ answers were noteworthy, and may be associated with their personal reservation in expressing themselves on this sensitive and intimate theme. To ensure diversity, inclusivity, and impartiality in nursing practice, it is imperative to change the curriculum plans of nursing courses to address the theme of sexuality during the training process of nurses in Europe.

## 1 Introduction

The relationship of societies and individuals with human sexuality has been undergoing major changes, influenced by technical and scientific knowledge, and demographic and epidemiological transitions (Manandhar et al., 2018).

Sexuality is a natural part of human development and a component of each individual’s personality. It includes several aspects such as sex, gender, gender identity, sexual orientation, eroticism, pleasure, intimacy, and reproduction (WHO, 2006). Sexuality is a subjective, dynamic, and complex experience influenced by biological, psychological, social, economic, political, cultural, legal, historical, religious, and spiritual factors (WHO, 2006; UNESCO, 2018), is a social construct shaped by cultural values, traditional beliefs, and social norms are expressed individually through thoughts, fantasies, desires, beliefs, values, behaviors, practices, roles, and relationships in various sociocultural contexts. Sexuality also refers to issues related to sexual and reproductive health, the use of technology, and the exercise of power in society (WHO, 2006; UNESCO, 2018). However, not all dimensions of sexuality are always experienced or expressed (WHO, 2006); some may be more evident than others and may be influenced by biopsychosocial processes that mark each stage of human development (UNESCO, 2018).

According to the World Health Organization (WHO), sex education for children and young people involves continuous learning about the cognitive, emotional, social, interactive, and physical aspects of sexuality, thus contributing to broader social changes, namely, incorporating education for citizenship and health (Picken, 2020).

The transition to higher education marks a stage in the lifecycle of young people with adjustments, changes, and multiple challenges. This transition is a path to professional achievement and social ascension, and promotes the expression of sexuality, new relational practices, and experiences driven by new academic experiences. Thus, the quality of sexual health of students in higher education acquires their own expressiveness. That is, along with the physical, emotional, and cognitive maturation changes to which young people are subjected to, there is exposure to situations of risk to sexual health resulting from risky behaviors, such as the consumption of alcohol, drugs, risky sexual practices (DGS, 2006; Firmeza et al., 2016), inadequate or absent guidance on contraceptive methods, and the prevention of sexually transmitted infections (STIs), with important emotional, organic, and socioeconomic repercussions (Moser et al., 2007).

In the search for full and secure sexuality and sexual well-being, sexual and gender diversity provide visibility to the broad and complex interaction of the various components of sexuality, namely, in the dimension of human freedom for any expression of affection and physical and emotional pleasure (WHO, 2006; UNESCO, 2018). By itself, the word sexuality has different meanings depending on the language, and when associated with strong sociocultural traits in the experience of sexuality, it is perceived that sexual behaviors are determined by diverse and sometimes distinct sociocultural norms of conduct between and within cultures (UNESCO, 2018).

Sexual and gender diversity imply knowledge of oneself in what one learns and incorporates throughout the process of change and maturation, desires and affections, behaviors, and ways in which each one presents oneself to others. It also requires broad information about concepts and terms related to the theme, awareness, and openness to the deconstruction of socially imposed and assumed prejudices and stereotypes, aiming at more inclusive societies where an egalitarian acceptance and respect for the other in their differences of identity and social role that they exercise, prevails.

International scientific evidence is consistent with the relevance of implementing student-centered sex education programs in schools adjusted to local ages and contexts (UNESCO, 2018). Educational programs offer knowledge and oriented information, positive and inclusive values, and skills to young people for a responsible practice of sexuality to make informed decisions about their sexual health (Mueller et al., 2008; Reis et al., 2011) and provide satisfaction, well-being, and quality of sexual life (DGS, 2006; UNAIDS, 2013; UNESCO, 2018). However, international technical guidelines on sex education target students aged 5–18 years (UNESCO, 2018; Cunha-Oliveira et al., 2021).

Thus, regarding sex education programs in the context of higher education, the evidence shows that they are especially centered on a biophysiological perspective, focused on the functioning of reproductive organs, devaluing sexual and gender diversity, and their practical and everyday needs (Gómez and Torres, 2015). Cunha-Oliveira et al. (2021), in theirs scoping review, on the approach to sex education from a gender perspective in youth education systems in Spain and Portugal, considering the legislation of both countries, concluded that the presence of an approach still to be consolidated, accompanied by political and legislative changes gradually implemented in both countries was based on the influence of the hegemonic social model: the patriarchal system. De Silva et al. (2019) added that, although the school environment contributes to changing the conception of sexuality, feelings such as shame and shyness persist in students, anchored in stereotypes and taboos. In a study of Portuguese nursing students, found a tendentiously conservative meaning in the role attributed to the female gender, resulting from the socialization process experienced where the role of family and religion were striking. Other studies on medical students and health professionals have reported that they did not receive sufficient education about sexual health and did not feel comfortable dealing with sexual problems (Beebe et al., 2021).

In the southern part of the European Union (EU), namely Spain, Italy, and Portugal, the teaching-learning process of nursing students advocates the acquisition of sexual competencies, through *“a standardized curricular dimension guided by a behaviorism based on a biological view of sexuality (...) approached from the perspective of reproductive health with scarce and outdated content that does not draw the attention of students”* (Soto-Fernández et al., 2023). A perspective that is not conducive, responsible, and healthy experience of one’s own sexuality, does not contribute to the training of competencies and skills to support and assist the sexuality of those cared for (Sehnem et al., 2013).

In fact, the literature clearly highlights the importance of creating guidelines and intervention strategies for the sex education of young people in higher education, which favors continuity in the acquisition of healthy skills, values, and behaviors. A holistic and inclusive approach includes a reflective practice on attitudes and behaviors (Sehnem et al., 2013; Soto-Fernández et al., 2023) appropriate to sexual and gender maturity and diversity, sociocultural contexts, and new decision-making possibilities promoting satisfactory sexuality (UNESCO, 2018). Considering the strong sociocultural and religious influence on sexuality issues, transnational and transcultural studies on sexuality and gender provide deeper knowledge about cultural diversity, contribute to the reduction of risk behaviors to sexual and reproductive health, as well as favoring the development of the cultural competence of health professionals and the cultural adaptation of sexual health promotion programs (Barroso, 2018; Alarcão et al., 2022). Highlighted here are aspects that contribute to achieving the Sustainable Development Goals of the 2030 Agenda, namely, continued progress in sexual and reproductive health - Goal 3, achieving gender equality and empowering all women and girls – Goal 5, and strengthening the implementation of partnership actions between countries for sustainable development - Goal 17 (United Nations, 2023).

The present study is part of a collaborative and multicenter project, entitled “Educating in Sexuality: Advancing European Health,” or “EdSeX,” with the main objective of describing the teaching of human sexuality in nursing courses and analyzing the context in which these contents are contemplated. Partner universities for this project are: The University of Castilla-La Mancha, Toledo, Faculty of Physiotherapy and Nursing, Spain; The University of Évora, Nursing School of S. João de Deus in Évora, Portugal; The Polytechnic Institute of Santarém, School of Health of Santarém, Portugal; The University of Modena and Reggio Emilia, Italy; and, as a guest university, Seattle University in the United States (Soto-Fernández et al., 2023).

The present study refers to one of four results generated in this multicentric project, namely Outcome 2 – Open Educational Resource (OER-EdSeX) for Higher Education, intended for students of the degree in nursing at the universities involved (Soto-Fernández et al., 2023) and aims to identify and analyze students’ knowledge about sexuality, sex, and gender diversity.

## 2 Materials and methods

This qualitative and descriptive study was a part of the workshop *OER-EdSeX, for Higher Education* (Soto-Fernández et al., 2023) as mention in the previous section.

Outcome 2 was organized into two training blocks, each of them subdivided into two modules: The 1st training module dealt with the main topic of *Covert Sexual Violence: Behind sexual consent?* and the 2nd training module addressed the topic of *Sexual Diversity: Validating emotions from sexuality.* The training blocks were developed in workshops and taught in the original language of each university, integrated into the project (Spanish, Portuguese, Italian, and English), with the intention of working on the foundations of healthy and holistic sexual education, with the student as the main protagonist and agent of health education. The EdSeX project predicted 10–30 participants for each partner university and 6 for the invited university. Nursing students were recruited through posters and in the classroom by a specialized faculty member (Soto-Fernández et al., 2023). The workshop was conducted between October and December 2022 in specific days, defined by each partner university, each with an approximate duration of 60–80 min.

With the aim of evaluating the knowledge obtained by nursing students who participated in the workshop, a questionnaire was prepared with the topics covered, since the questionnaire is a written recording instrument, properly planned to research the subject’s data, through questions, referring to knowledge, attitudes, beliefs, and feelings (Vilelas, 2022). This way, data collection was performed through a mixed questionnaire with a combination of closed and open-ended responses, allowing free responses to the variables under study. The questionnaire was organized into three parts: (1) the first contained sociodemographic variables; (2) the second included questions assessing cognition, opinion (suggesting exemplification of a specific situation) and students’ attitudes regarding the topics of sexuality, sex and gender diversity; (3) and the third part, focused on the domain of behavior/attitudes with statements of myths and realities about sexuality.

Participating nursing students completed the questionnaire after the workshop. The students were informed of the nature of the study, their time commitment to participate, and that there were no consequences for not participating. Written informed consent was obtained from all participants. The average response time of the questionnaire was approximately 15 min.

A total of 75 students from the 2nd, 3rd, and 4th year of the nursing degree were recruited through convenient sampling, which was representative of students from the Faculty of Physiotherapy and Nursing in Toledo, Spain; the Nursing School of S. João de Deus in Évora; and the Health School of Santarém in Portugal. Only students of 2nd, 3rd, and 4th year, were recruited since, the 1st year students would have had either little or no sexuality content or would not have started taking nursing classes yet. Students from the Universities of Modena and Reggio Emilia and Seattle Pacific University, as foreseen in the initial project, did not complete the questionnaire after the workshop; therefore, they were not included in this study.

### 2.1 Ethical considerations

This study was part of a multicenter research, and followed the Declaration of Helsinki. It was submitted and approved by the Ethics Committee of Social Research of the University of Castilla-La Mancha, Toledo, Spain (CAU-661803-V4Z4). All participants were informed about the nature of the study, and provided written informed consent.

### 2.2 Data analysis

Sociodemographic data and other closed questions were analyzed thru Excel® version 20 and the IRaMuTeQ Software (Interface de R pour les Analyses Multidimensionnelles de Textes et de Questionnaires) 0.7 alpha 2, which allows statistical analyses on textual corpus and on individual/word tables with the advantage of encoding, organizing, and separating information (19), was used for the textual data of the open questions (Camargo and Justo, 2018).A *textual corpus* is a set of texts to be analyzed (Camargo and Justo, 2018); in this case, it was applied to the questionnaire responses of nursing students.

For the analysis of the *textual corpus* of the present investigation, the Descending Hierarchical Classification (DHC) of the Reinert method was used. The text segments were classified according to their respective vocabularies, the set was distributed based on the frequency of reduced forms, and organized in an easily understandable and visually clear manner (Camargo and Justo, 2013; Camargo and Justo, 2018) through the grouping of statistically significant words and their qualitative analysis. Each interview was called an Initial Context Unit (ICU). The Elementary Context Units (ECU), in turn, correspond to the text segments that make up each class and are obtained from the ICU, presenting vocabularies similar to each other and different from the ECU of the other classes (Camargo and Justo, 2013; Camargo and Justo, 2018).

The present analysis of the *textual corpus* was carried out in 4 stages: configuration of the *textual corpus* to be analyzed through the transcription of the written questionnaires, complying with the procedures defined by the IRaMuTeqQ software; importation of the *textual corpus* of analysis; encoding of the initial text through its processing by DHC techniques; and the interpretation of the generated classes based on the dictionary of words and the use of the chi-square (χ^2^) (Camargo and Justo, 2018).

After transcribing and reading the answers to the questionnaires, an analytical model composed of categories was built that corresponded to the word classes generated by IRaMuTeQ. Categories can be established as priori in the exploratory phase of the research or as posteriori after the research has been carried out (Bardin, 2016). In this study, we established *a posteriori* analytical category, which was analyzed and interpreted using the thematic analysis technique of the content analysis method (Bardin, 2016; Vilelas, 2022).

## 3 Results

**Figure 1:**
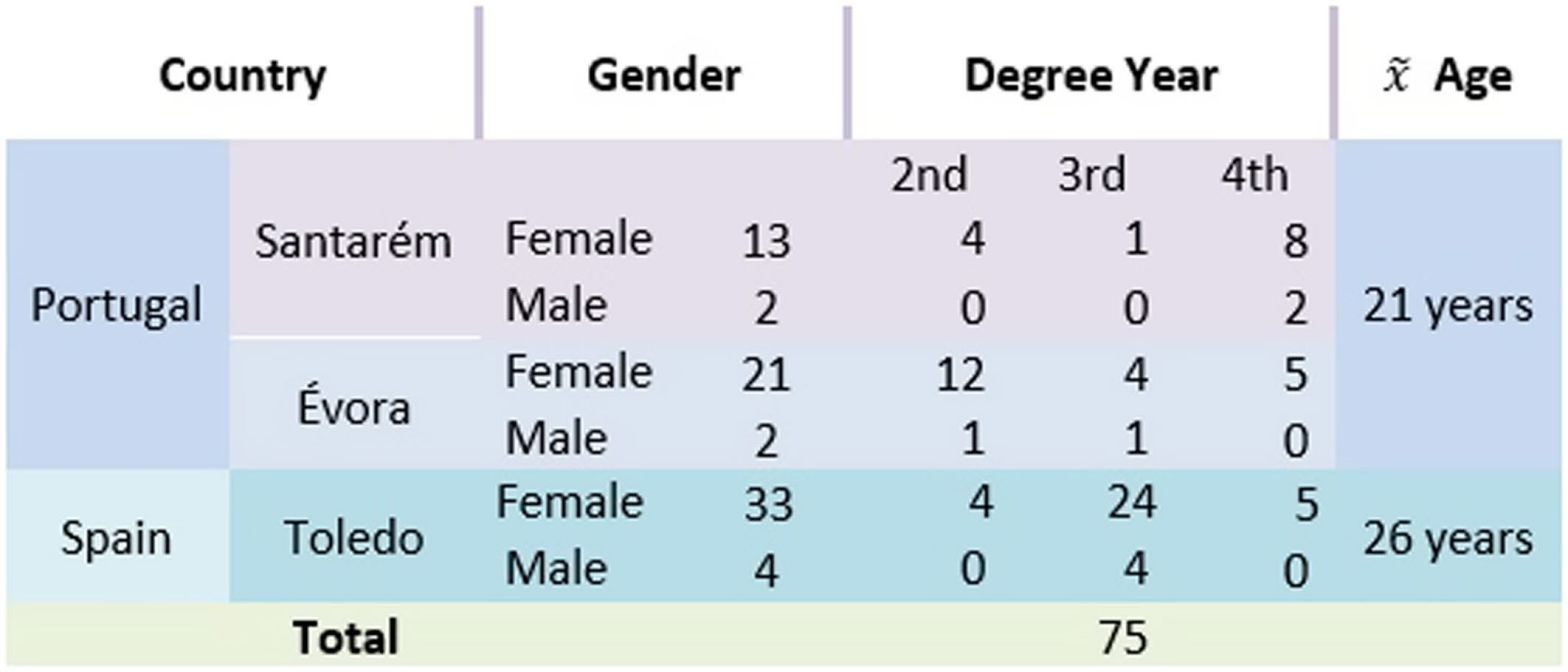
Demographic distribution of students by country, year degree, and gender.

The sample consisted of nursing students (*n* = 75) from the two countries included in the multicenter study (Spain and Portugal), with an average age of 26 and 21 years respectively, attending the 2nd (*n* = 21), 3rd (*n* = 34), and 4th (*n* = 19) years of the program (Figure 1). The majority were female (89.3%), reflecting the trend at EU level with regard to students enrolled in higher education as can be seen in the 2021 statistical data, by teaching area, with women (72.0%) are mostly enrolled in the area of Health and Social Protection (Fundação Francisco Manuel dos Santos and Pordata, 2023). Nursing is a career widely chosen by women, associated with its nature of caring and imparts social attributes associated with femininity, generating prejudices in populations and in students’ choices, namely the risk of being devalued professionally (Sim-Sim et al., 2022).

The *textual corpus* consisted of 75 ICU, corresponding to all applied questionnaires, and 312 ECU of which 260 text segments were used, which corresponds to 83.33% of the total textual corpus. An index of 75% or more is considered a good use of ECU (Camargo and Justo, 2018; de Souza et al., 2018).

**Figure 2:**
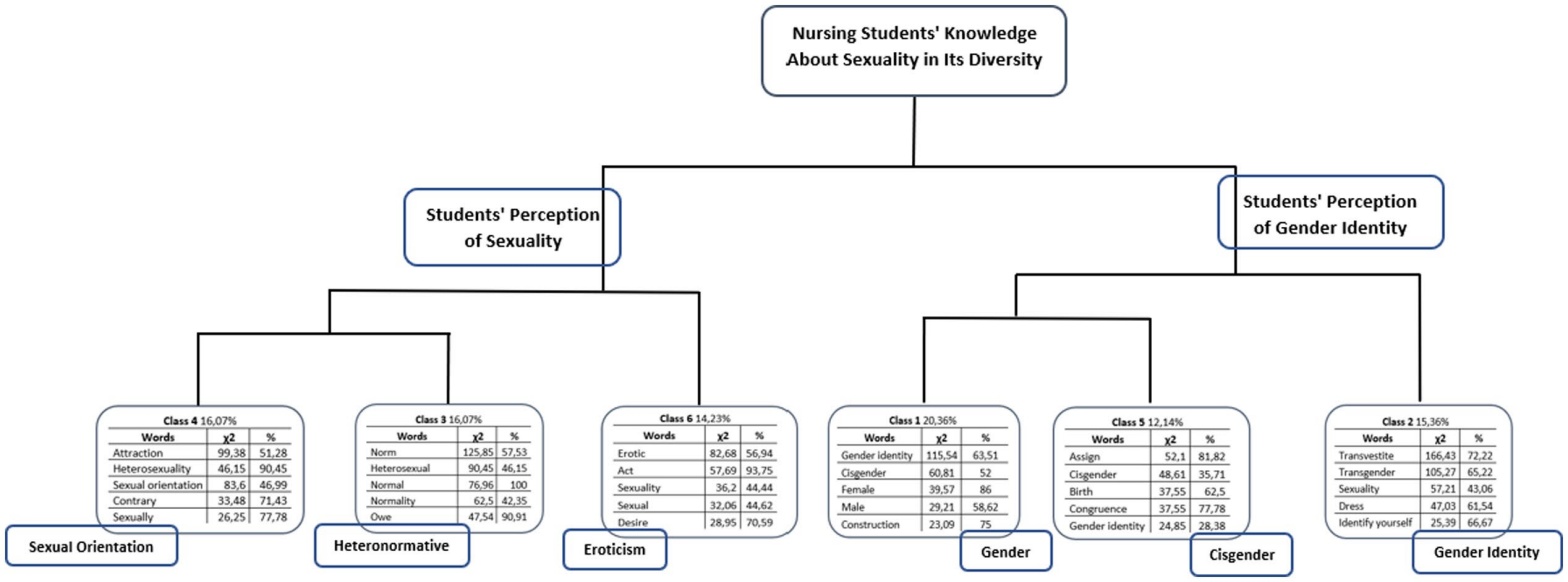
Dendrogram of descending hierarchical classification with partitions and research corpus content. Source: research data from IRaMuTeQ.

From these ECUs, of the DHCs, six classes emerged, which represent the segments of the text classified according to their respective vocabularies, considering the analysis of the words that presented a χ^2^ value greater than 3.84 and *p* < 0.0001, aiming at its significance and reliability (Camargo and Justo, 2013; Camargo and Justo, 2018; de Souza et al., 2018) (Figure 2).

Figure 2 illustrates the DHC dendrogram, allowing us to understand the expressions and each of the words enunciated by the participants, analyzing them from their places and social insertions (Camargo and Justo, 2018). Reading from left to right, it presents the distribution moments that were conducted in the *textual corpus* and their relationships until the final classes emerged. In the first distribution, the *textual corpus “Nursing students’ knowledge about sexuality in its diversity,”* was divided into two sub-corpuses: *“Students’ perception of sexuality”* and *“Students’ perception of gender identity.”* In a second instance, both sub-corpuses were subdivided into tree others, originating Class 6 “Eroticism” (14.23%), Class 4 “Sexual Orientation” (16.07%) and Class 3 “Heteronormative” (16.07%). Classes 3 and 4 have a greater proximity to each other and consequently to Class 6. Similarly, Class1 “Gender” (20.36%) and Class 5 “Cisgenders” (12.14%) also presented a greater interrelationship between themselves and consecutively with Class 2 “Gender Identity” (15.36%). The DHC ended here with the stabilization of the six classes composed of ECU with similar vocabulary, which is the result of a selection carried out automatically by the software, thus avoiding any bias of the researchers in the selection of contents. Data from emerging classes/categories were interpreted using the thematic analysis technique of content analysis (Bardin, 2016). Content analysis is a set of communications analysis techniques, through systematic and objective procedures for describing the content of messages. Often used as a diagnostic tool, in order to make specific inferences or causal interpretations about a given aspect to be investigated, based on the frequency of appearance of certain elements of the message (Bardin, 2016).

## 4 Discussion

Based on the objective of this study to identify and analyze students’ knowledge about sexuality, and sexual diversity, we present an interpretative analysis of the *textual corpus*: *“Nursing students’ knowledge about sexuality in its diversity.”*

### 4.1 Students’ perception of sexuality

The sub-corpus *students’ perception of sexuality* comprised classes/categories pertaining to knowledge about the concepts of sex, sexuality, sexual orientation, heterosexuality, and eroticism. From these emerged Class 4 “Sexual Orientation” (16.07%), Class 3 “Heteronormative” (16.07%) and, Class 6 “Eroticism” (14.23%).

Class 6 (14.23%), farther from the rest, represented the students’ perception of the concept of sex, through the expression of eroticism, through words, erotic (56.94%, χ^2^82.68), act (93.75%, χ^2^57.69), sexuality (44.44%, χ^2^36.2), sexual (44.62%, χ^2^32.06), and desire (70.59%, χ^2^28.95). Although sex refers to anatomical and other biological differences between men and women that are determined at the time of conception and intrauterine development (Rubin et al., 2019; Costa-Val et al., 2023; Ludwig et al., 2023), not all students have this understanding of the concept.

*Biological dimension that includes the external and internal sexual organs, chromosomes and basically, is the sexual act (P7).*

*It is the act performed by two people who relate and act for pleasure or for reproduction (P19).*

*On the one hand, I understand sex referring to the biological, I mean, if the reproductive system you are born with is female (vagina) or male (penis). On the other hand, I understand sex as the sexual act between two or more people (P32).*

Most students understood the concept of sex as only a physical act or sexual intercourse. Sexual acts and sexual activity are distinct concepts with different dimensions, although they are difficult to define because of their close relationship. Some studies have highlighted that people distinguish between having sex and sexual activity. Sexual activities included practices such as individual masturbation, cybersex, and oral sex (Halwani, 2023). The students’ responses demonstrated the difficulty of defining the concept.

*Sexual act and physical contact between two people (P66).*

*It is the expression of your sexuality either as a couple or with yourself (P30).*

Sex also appeared to be associated with eroticism, as we can see from the proximity of the prevalence of words in students’ responses.

*Stimulus awakened in people, through the five senses, that is, without having sexual intercourse itself. It’s like what happens in the seduction phase or demonstration of interest (P3).*

*Erotic, is related to sexual and intimate relationships. It has to do with sexual attractions, which cause, for example, pleasure (...) sexual desire (P39).*

Eroticism, which is also difficult to define (Fellmann, 2016), is not just a sexual activity; it is a psychological aspect dependent on and, at the same time, independent of sexuality. Throughout human evolution, erotics have diverted sexual activity from the main goal of reproduction (Fellmann, 2016; Halwani, 2023). For students, these concepts of sex and eroticism are more closely related to desire, pleasure, and effective sexual acts than to the full experience of sexuality in itself.

*Sensuality, fantasy. What is meant by erotic* var*ies from person to person (P5).*

*Eroticism is given to those who make us receptive and to those who give us pleasure in an individual and unique way (P51).*

*There are several forms of pleasure, such as kissing, caressing, hugging, sexual intercourse (…). Also including non-physical forms (P56).*

*It is the attitude and desire in sexual activity (P75).*

Within this sub-corpus, Class 4 (16.07%) and Class 3 (16.07%) appear, representing the concepts of “sexual orientation” and “heteronormativity,” associating the concept of heterosexuality with the social norm, as happens in some countries of the EU (Michielsen and Ivanova, 2022). In Class 4, words such as attraction (51.28%, χ^2^99.38), contrary (71.43%, χ^2^33.48), sexual orientation (46.99% χ^2^83.60), and heterosexuality (90.45%, χ^2^46.15) significantly identified heterosexuality as a sexual orientation, demonstrating students’ knowledge of the concept.

Currently, sexual orientation is understood as the basic and organizing sexual preferences of one person over another (Dalrymple et al., 2020; Halwani, 2023). Three main components of sexual orientation can change throughout one’s life cycle: attraction, behavior, and identity. Some of the most common sexual orientation identities are lesbian, gay, bisexual, and straight, although many more (Dalrymple et al., 2020) are represented by the acronym LGBTQIA+. Nursing students demonstrated knowledge of this concept.

*Each person’s sexual orientation depends on their attraction to one gender or another, although it can also be for both or even for neither (P28).*

*Sexual orientation is associated with what the person means and what they are attracted to (P66).*

*It is the gender that the person is attracted to according to their sexual identity (P67).*

As previously mentioned, heterosexuality is a sexual orientation identity; thus, understanding the proximity of Classes 4 and 3. The concept of heterosexuality refers to the attraction of a person to another of the opposite sex, although the strictest definition states attraction to the opposite gender (O’Brien, 2012). Regarding this concept, students demonstrated knowledge of both simple and strict aspects, with no uniformity of answers.

*By heterosexual I mean a person whose sexual orientation states that you are attracted to a person of the opposite sex (P27).*

*Person who is attracted to people of a different gender (P33).*

*Those people who like others of the opposite gender (P44).*

*It is a person attracted to the opposite sex (P55).*

Currently, we live in a society that is constantly changing, but heterosexuality remains largely represented by various forms of artistic and social expression (O’Brien, 2012; Brancaleoni et al., 2018), leading students to assume that this is the current social norm.

*Heterosexuality is the norm because it allows reproduction and therefore is what is most “accepted” by society (P15).*

*Culturally and socially, it is “accepted” (P50).*

Heteronormativity can be understood as the invisible deductive imposition of a normative binary framework of sex and gender and the practice of coercive and naturalized heterosexuality in societies worldwide (O’Brien, 2012; Wilkinson, 2023). Although 60% (*n* = 45) of the students stated that heterosexuality is not a social norm, it is considered “*more socially accepted,*” justifying the approximation of these classes.

*It is what has classically been socially accepted, but it is not the norm (P28).*

*It’s not the norm. It’s the most common, but it does not have to be that way. It’s like that because that’s how we were brought up (P49).*

*It’s not the norm. I believe it still makes up most of the cases in the country. It was what was always transmitted by ancient generations by culture, as well as by the church and being a country based on Christianity, it has always influenced people’s way of thinking (P55).*

It should be noted that, despite being able to define heterosexuality, 12% of the participants did not respond when asked about their understanding of whether they “think it is the norm.” This lack of response may demonstrate not a lack of knowledge, but an omission of your personal and intimate opinion on the issue. This attitude can be understood as the acceptance of this norm or, in contrast, its non-acceptance.

**Figure 3:**
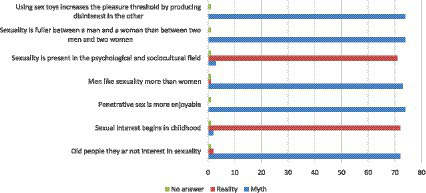
Myths and beliefs about sexuality.

Still, regarding the students’ perception of human sexuality, they were presented with several statements about specific situations alluding to the theme, for them to classify the statements as myths or reality present in today’s society. After the statistical analysis of the data, it was identified that the majority (96%) were able to recognize statements called myths and distinguish them from reality statements. Only one student did not answer this question (1.33%), as shown in Figure 3.

Through the analysis of the dendrogram, the students’ answers verified the existence of knowledge about some concepts referring to sexuality, namely, the prevalence of heterosexuality in the context of sexual orientation, bringing it closer to the current social normative standard, although agreement with the same was not expressed in their responses.

### 4.2 Perceptions of gender and gender identity

The sub-corpus *perception of gender and gender identity* is representative of the recognition that the student has of himself, within the standards of female and male gender, in an understanding that integrates the social, cultural, and historical processes that determine and link gender (Manandhar et al., 2018) and a gender identity that refers to the experience of gender felt individually, which may or may not be in accordance with the physiology of the person or the sex assigned at birth (UNICEF, 2017; Manandhar et al., 2018; WHO, 2021).

In this sub-corpus, from the expressiveness of the words in the corpus of analysis, the classes/categories emerged: Class 1 “Gender,” Class 2 “Gender identity,” and Class 5 “Cisgender.”

Class 1, the most expressive, with a classification of 20.36%, showed how the concept of gender was understood by the students. This was mostly described as a binary construction (75%, χ^2^23.09) about the sexual differences that socially reference the feminine and the masculine; which according to Basso and Paula (2020) among the classifications of sexual gender, gender binary is the most common way to determine gender.

*How society classifies you for having the genitals that you have (sex) (P31).*

Despite a significant number of responses with brief descriptions of the concept of gender, a characterization that includes socialization, norms, and gender roles stands out (Heise et al., 2019). However, since gender is based on sexual bodies, the biological dimension was also considered by the students.

*Gender is a social label (P18).*

*Gender is a social construction, namely the sum of values, attitudes, roles, practices, or cultural characteristics, based on sex (P6).*

The manner in which gender is expressed is revealed in the description of its concept. However, under the effect of social pressure, culturally determined attributes and rights for women and men (which vary from culture to culture) are identified simultaneously with power relations between them (Manandhar et al., 2018).

*They are those behaviors or attributes that are considered to be male or female (P21).*

*Gender is the set of expectations, behaviors, and rules that are expected of people, and are very diverse (P43).*

However, in an opposite sense, for some students, the concept of gender still represents the biological dimension, that is, as a synonym of “sex,” referring to what is proper to the male and female sex. According to Heise et al. (2019), these two terms are often confused not only in popular discourse, but also in the scientific literature on health.

*Gender has to do with the biological side, with the sexual organs (*P57*).*

The terms gender identity (63.51%, χ^2^115.54) and cisgender (52%, χ^2^60.81) being expressive in this class, not only reveal a close relationship between the concepts, but also reflect an imprecise description of the concept of gender, with some incongruity in its definition.

*Gender is how I feel, whether I identify more as female or male (P53).*

Class 2 “Gender Identity,” with a percentage of 15.36% (χ^2^115.54), revealed the main aspects that give meaning to gender identity. Gender identity is the inner feeling of being a woman, a man, another gender, or not having any gender, which may not align with the sex assigned at birth and may change over time for some people (Dalrymple et al., 2020). In the students’ answers, the way a person builds and recognizes himself is described with expressions that give meaning to the perception they have of their gender, valuing their individual character.

*Gender identity is how you really feel (P20).*

*Gender identity is based on which one feels most identified (P25).*

*Gender identity is how I see and feel in my own body (P58).*

The data suggest the existence of a concerted understanding that all people have a gender identity, in the way each one sees himself, thinks and wants to be socially recognized, represented by the word “Identify yourself” (66.67%, χ^2^ 25.39). Consistency in recognizing that not all people fit into the binary notion of man/woman by reference to different perceptions of gender, namely that of an identity associated with biological sex (cisgender) or of the person who identifies and/or expresses your gender identity different from the gender you were assigned at birth (transgender).

*It’s how you identify; it does not have to agree with your gender at birth (P23).*

*A person can be female and identify as male (P11).*

*Gender identity is how a person feels. It is the perception of gender; the woman, man or combination of both; internal reference mark (P47).*

The expression of terms such as “Transvestite” (72.22%, χ^2^166.43), “Transgender” (65.22%, χ^2^105.27) and “Clothing” (61.54%, χ^2^47.03) in Class 2 are suggestive that the students had knowledge that allowed them to distinguish terms that define different gender identities. Transgender is defined as a person whose gender identity does not correspond to the biological sex assigned at birth (Basso and Paula, 2020; Cislaghi and Heise, 2020) and who expresses, behaves, or assumes social roles different from those socially agreed upon for their gender (Basso and Paula, 2020). It encompasses different types of gender identities such as transsexuals, transvestites, queers, and bigender, among others (Basso and Paula, 2020). With regard to the definition of the terms that were questioned–transgender, transsexuals, and transvestite, even after approaching them in a workshop, there were descriptions with reference to one or two characterizing aspects of each term, suggestive of a “popular” knowledge about these unconventional gender identities. When other characteristic aspects were mentioned, they appeared in an inaccurate or incongruous way, suggesting a weak representation of each of these words, maintaining a very close relationship with the definition of transgender.

In the description of the transvestite, the person who is born male and has a female gender identity is globally referenced, and very often the students refer to the fact that they dress in women’s clothes (clothing). It was described with some frequency as assuming gender roles that were different from those determined by society. However, the possible changes in the body are not very visible, and in this type of gender identity, one does not want to undergo surgery for sexual reassignment.

*A person, usually a man, who presents and expresses himself as the opposite sex, however, identifies with his birth sex (P1).*

*Man, who wears clothes and behaves like a woman (P12).*

*Person who dresses in a way contrary to what society dictates (P18).*

Class 5 “Cisgender,” with a percentage of 15.36%, defined as a person whose gender identity is congruent with the identity associated with their biological sex and/or social designation (Basso and Paula, 2020) was a term that students defined correctly and precisely, by the expressiveness of the words “Attributed” (81.82%, χ^2^52.10), “Congruence” (77.78%, χ^2^37.55) and “Birth” (62.50%, χ^2^37.55) associated with this class. However, this may also suggest a greater willingness to expose a socially accepted gender identity, and, simultaneously, protection, meeting the evidence that shows that people who deviate from prevailing gender expectations can suffer discrimination and social sanctions, namely the stigma associated with mental illness, disrespecting their freedom and dignity (Castro-Peraza et al., 2019).

*It is the coherence and identification of a person who in childhood was designated as a woman, having feminine characteristics and who identifies as a woman (P2).*

It should also be highlighted that the influence of social values in the construction of gender identity was not consistent in the students’ responses, and the cultural influences and historical contexts in which each person is located were not mentioned as characteristics of this concept. The study by Sim-Sim et al. (2022) reveals that students from conservative religious backgrounds are more traditionalist in their attitudes towards sexuality.

In fact, despite a progressive change, both in the educational and legislative systems, namely in Spain and Portugal (Cunha-Oliveira et al., 2021), and the progressive emancipatory claims of transgender people and people with gender diversity drawing attention on a global scale, they together show restrictive societies where gender inequality is implicit, (Heise et al., 2019; Cislaghi and Heise, 2020) and manifests itself in different ways depending on the social and cultural models present in societies. Furthermore, scientific evidence has demonstrated the absence of LGBTQIA+ population from health services and consequently more pathologies due to the lack of preparation of health professionals (de Moura et al., 2023).

## 5 Conclusion

Nurses constitute one of the largest professional groups in the field of health, and consequently, have a greater relationship of proximity and responsibility with people in their health-disease process. In this way, they are in a privileged position to act as catalysts for change towards quality health care, sensitive to sexuality issues, not in line with socially determined behaviors for men and women, but with culturally competent behaviors in sexual diversity, (Heise et al., 2019) and as promoters of sexual and reproductive health through education and awareness (Michielsen and Ivanova, 2022).

In 2020, the WHO suggested a fundamental role for higher education in Europe’s future. Sexuality emerges as a relevant programmatic content to be implemented in nursing training (Picken, 2020), giving continuity to the “school” as an institution that can influence and contribute to the process of building an inclusive education with regard to sexual and gender diversity, demystifying inequalities and respecting individuality (Rodrigues, 2017). Simultaneously, developing nursing students’ knowledge of themselves, their references, values, beliefs, and (pre)concepts, and, in a professional way, developing interpersonal and systemic skills to address human sexuality in nursing care throughout the life cycle, integrating sociocultural, historical, and political influences, is important. However, literature has shown that the approach to sexuality is still incomplete in curriculum plans (Cunha-Oliveira et al., 2021; Patrão et al., 2023; Soto-Fernández et al., 2023).

The study aims to show the teaching community the importance of integrating content related to sexuality and health education into students’ curricula. The results revealed that the nursing students participating in the study, after holding a workshop on the theme of sexual diversity, when asked to describe terms related to sexual orientation, gender diversity, and sex, their opinions and examples demonstrated only elementary knowledge, not allowing the appropriation that characterizes and differentiates each of the terms. The study suggests that the difficulty in assuming a personal position in relation to a sensitive, intimate theme that is marked by a sociocultural construction may be underlying, namely, when verifying the frequent use of the third person (she/his/she or the person) in the answers given. A significant aspect of the professional practice of nursing is that it focuses on the interpersonal relationship of the nurse with the person/group of people (clients of care), and is ruled by humanistic principles, respect for freedom, human dignity, and the values of people/groups (Consejo General de Enfermería, 1998; Ordem dos Enfermeiros, 2015).

Therefore, it is fundamental to reflect on and change the curriculum plans of nursing courses through the inclusion of the theme of sexuality in a comprehensive, impartial, and inclusive manner. For future nurses to provide quality, person-centered sexual and reproductive healthcare, knowledge supported by scientific evidence is essential, thus enabling the removal of stereotypes and socially rooted prejudices that can lead to stigmatization and have a negative impact on people’s health.

To verify a change in the practice of nursing care, it is also relevant to develop the skills and knowledge of teaching staff regarding sexual health beyond the biological, thus integrating psycho-emotional dimensions. Changes must also be driven by an active role with peers and nurses in clinical practice. Health professionals mention this flaw in their curricula and the need for better skills to work in the sexual health care of the general population and the LGBTQIA+ population, particularly in overcoming prejudice and resistance (Costa-Val et al., 2023).

The EdSeX project aims to improve this knowledge by promoting up-to-date educational strategies and tools suitable for the different populations where it operates. Although the study sample was a limitation, as it included only 75 participants. However, we consider that the study was very important and made a significant contribution to Outcome 2 – Creation of the Open Educational Resource (OER-EdSeX) for Higher Education, with the specific objective of profiling students’ attitudes and beliefs about sexuality and sex education (Soto-Fernández et al., 2023), favoring the implementation of joint transformative strategies and actions, in line with sustainable development goals 3, 5, and 17 of the 2030 Agenda.

## Data availability statement

The original contributions presented in the study are included in the article/supplementary material, further inquiries can be directed to the corresponding author.

## Ethics statement

The studies involving humans were approved by Ethics Committee of Social Research of the University of 165 Castilla-La Mancha, Toledo, Spain (CAU-661803-V4Z4). The studies were conducted in accordance with the local legislation and institutional requirements. The participants provided their written informed consent to participate in this study.

## Author contributions

CS: Software, Resources, Methodology, Data curation, Conceptualization, Writing – original draft, Investigation, Formal analysis. AG: Formal analysis, Investigation, Writing – original draft, Conceptualization, Data curation, Methodology, Resources, Software. TC: Validation, Supervision, Data curation, Writing – review & editing. SP: Validation, Supervision, Data curation, Writing – review & editing. FB: Validation, Supervision, Writing – review & editing, Data curation. JP-P: Visualization, Writing – review & editing, Data curation. AF: Validation, Supervision, Project administration, Writing – review & editing, Conceptualization. SG-C: Validation, Supervision, Project administration, Funding acquisition, Writing – review & editing, Conceptualization. HD: Validation, Supervision, Project administration, Writing – review & editing, Conceptualization.
